# Surprise disrupts cognition via a fronto-basal ganglia suppressive mechanism

**DOI:** 10.1038/ncomms11195

**Published:** 2016-04-18

**Authors:** Jan R. Wessel, Ned Jenkinson, John-Stuart Brittain, Sarah H. E. M. Voets, Tipu Z. Aziz, Adam R. Aron

**Affiliations:** 1 Department of Psychological and Brain Sciences, College of Liberal Arts and Sciences, University of Iowa, Iowa City, Iowa 52245, USA; 2 Department of Neurology, Carver College of Medicine, University of Iowa, Iowa City, Iowa 52245, USA; 3 Psychology Department, University of California, San Diego, La Jolla, California 92093, USA; 4 Nuffield Department of Clinical Neurosciences, John Radcliffe Hospital, University of Oxford, Oxford OX1 2JD, UK; 5 School of Sport, Exercise and Rehabilitation Sciences, University of Birmingham, Birmingham B15 2TT, UK; 6 Nuffield Department of Clinical Neurosurgery, John Radcliffe Hospital, University of Oxford, Oxford OX1 2JD, UK

## Abstract

Surprising events markedly affect behaviour and cognition, yet the underlying mechanism is unclear. Surprise recruits a brain mechanism that globally suppresses motor activity, ostensibly via the subthalamic nucleus (STN) of the basal ganglia. Here, we tested whether this suppressive mechanism extends beyond skeletomotor suppression and also affects cognition (here, verbal working memory, WM). We recorded scalp-EEG (electrophysiology) in healthy participants and STN local field potentials in Parkinson's patients during a task in which surprise disrupted WM. For scalp-EEG, surprising events engage the same independent neural signal component that indexes action stopping in a stop-signal task. Importantly, the degree of this recruitment mediates surprise-related WM decrements. Intracranially, STN activity is also increased post surprise, especially when WM is interrupted. These results suggest that surprise interrupts cognition via the same fronto-basal ganglia mechanism that interrupts action. This motivates a new neural theory of how cognition is interrupted, and how distraction arises after surprising events.

## Abstract (web summary)

Surprising events affect ongoing behaviour and cognitive processing, yet the underlying neural mechanisms remain unclear. Wessel and colleagues show that surprise recruits a motor suppression mechanism which may be implemented via the sub-thalamic nucleus and interrupts working memory performance.

## Graphical abstract

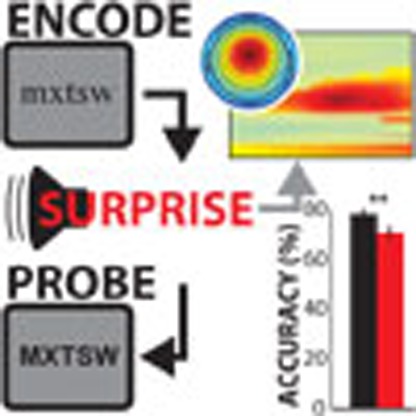

Surprising events often interrupt our ongoing train of thought and lead to forgetting. By what mechanism does this happen? Here, we test the hypothesis that surprising events recruit a brain system for widespread suppression of both ongoing motor and cognitive representations. We suppose this brain system is the same one that is recruited by rapid motor stopping, which implicates a fronto-basal ganglia network including the pre-supplementary motor area^1^,^2^, the right inferior frontal cortex^3^,^4^,^5^,^6^ and the subthalamic nucleus (STN) of the basal ganglia^7^,^8^,^9^. The hypothesis that surprising events recruit this same system to interrupt cognition (in this case, working memory (WM)) is motivated by several observations.

First, a recent study^10^ showed that when surprising events occur during a reaction time task, they induce motor slowing by recruiting the same neural suppressive neural mechanism that is recruited by outright motor stopping in a stop-signal task (SST^11^). Second, the neural suppressive mechanism for outright motor stopping in the SST has a broad effect on the skeletomotor system: stopping one motor effector in the SST leads to reductions of corticomotor excitability in other, task-unrelated effectors, even those that are at rest^12^,^13^,^14^,^15^. Moreover, such broad skeletomotor effects also occur after surprising events, consistent with the fact that surprising events recruit the same suppressive system as outright motor stopping^10^. Third, recent behavioural studies have shown that motor stopping has effects even beyond the motor system, as it affects stimulus value^16^ and WM encoding^17^. On this basis, we surmise that the impact of surprising events on cognition could relate, at least partially, to the same broad neural suppressive mechanism that is actively recruited to interrupt motor activity (e.g., in the SST).

To test this hypothesis, we developed a task in which WM maintenance was occasionally interrupted by surprising events (Fig. 1a). On each trial, participants encoded a string of consonants into WM, maintained it across a delay, and were then tested with a probe. Importantly, the WM probe was preceded by a sound. On 80% of trials, a standard sine-wave tone was played, with which the participants were familiar from practicing the task before the main experiment. However, in 20% of trials of the main experiment, a surprising birdsong segment was played instead of the standard tone. Behaviourally, we predicted that WM accuracy would be reduced following surprising tones. We further predicted that tones leading to WM failures would be more surprising than tones that were followed by correct WM probes.

We then tested whether the recruitment of the neural suppressive mechanism underlying motor stopping could explain WM failures following surprising events. Activity of this neural suppressive mechanism can be assayed using scalp electrophysiology (EEG) and local field potential (LFP) recordings from structures deeper in the brain.

On the scalp, motor stopping is indexed by a fronto-central slow-wave EEG signal component, whose timing correlates strongly with the speed and success of stopping^18^, and whose neural generators are independent of attentional or signal detection processes^19^. We aimed to isolate this fronto-central component by comparing successful to failed stop trials in an SST (which was performed by each participant after completion of the WM task). We predicted that surprising events in the WM task would recruit this same fronto-central EEG signal component, and moreover, that the recruitment of this component would be in proportion with the degree of WM suppression (i.e., greater surprise-related activity in the brain system indexing successful motor suppression would coincide with greater WM suppression).

Intracranially, motor stopping is indexed by depth electrode recordings from the STN in Parkinson's disease patients: STN activity is increased on successful versus failed stop trials^9^,^20^,^21^. The STN specifically is thought to be a key node in the wider stopping system^22^, and is perhaps responsible for the broad motor suppression that has been observed in both the SST and following surprise. This influence of the STN may putatively extend to the cognitive domain, thereby interrupting WM. Hence, we predicted that surprising events in the WM task would lead to increased STN activity. Furthermore, we predicted that increases in surprise-related STN activity would correspond with more WM failures.

Our results show that surprising events disrupt ongoing WM, leading to lower accuracy on subsequent probes compared with non-surprising events. Furthermore, very surprising events lead to greater accuracy deficits than less surprising events. Our scalp-EEG data show that surprise recruits the same independent neural signal component that indexes action stopping in the stop-signal task. Crucially, single-trial analyses show that low-frequency activity of this brain mechanism mediates the decremental effect of surprise on WM accuracy. Finally, the STN-LFP data show that the STN is also recruited following surprising events, and that its activity is greater the stronger the negative impact of surprising events on WM. Together, these results suggest that surprise interrupts cognition via the same fronto-basal ganglia mechanism that interrupts action.

## Results

### Behavioural experiment

**Figure 1:**
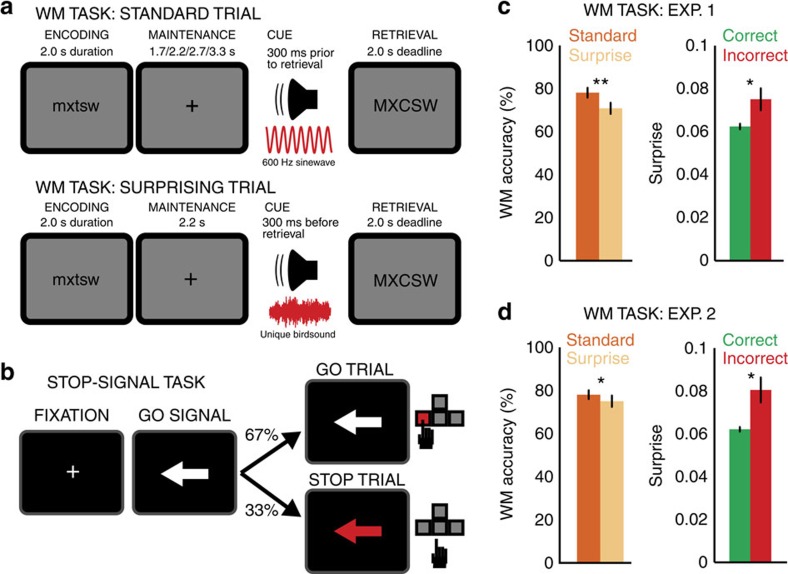
Behavioural task details and results. (**a**) WM task diagram. (**b**) Stop-signal task diagram. (**c**) WM task, behavioural data from the behavioural experiment. Left panel: WM accuracy by trial type. WM accuracy is reduced following surprising compared with standard tones (paired samples *t*-test, *N*=20, *t*(19)=3.5, *P*=0.0026 , *d*=0.78). Right panel: Bayesian surprise values of surprising trials split by WM accuracy. Surprise is increased for tones that interrupted WM (paired samples *t*-test, *N*=20, *t*(19)=2.14, *P*=0.045, *d*=0.77). Error bars denote s.e.m. (**d**) WM task, behavioural data from the scalp-EEG experiment, description as in **c** (WM accuracy: paired samples *t*-test, *N*=20, *t*(19)=2.3, *P*=0.033 , *d*=0.49; increased surprise for failed WM: paired samples *t*-test, *N*=20, *t*(19)=2.6, *P*=0.019, *d*=0.91).

Twenty healthy volunteers performed the WM task. On each trial, they encoded a letter string and held it across a variable delay period, the end of which was announced by either a standard or novel tone. Three-hundred millisecond after the tone, participants were probed for WM accuracy. As predicted, WM accuracy was reduced followed surprising compared with standard tones, with medium-to-large effect size (paired samples *t*-test, *N*=20, *t*(19)=3.5, *P*=0.0026, *d*=0.78, Fig. 1c). WM accuracy for the 2,200 ms interval was 77.9% for standard tones and 71.4% for novel tones; the significance test was based on that interval (WM accuracy for the three other intervals, which had standard tones only, was 75.1% for 1,700 ms, 76.5% for 2,700 ms and 75.3% for 3,200 ms). Furthermore, this reduction was related to the degree of surprise. The fact that the level of surprise wore off as the task progressed (even though all birdsong segments were unique) allowed us to model the effect of highly surprising compared with less surprising events. The level of surprise was quantified trial-to-trial using a Bayesian algorithm that compared the posterior probability of a surprising tone (i.e., the probability of a surprising tone on all trials up onto and including the current trial) with the prior expectation of a surprising tone (i.e., the probability of a surprising tone on all trials up onto – but excluding – the current trial) using the Kullback–Leibler divergence (see Methods section). Based on the model's surprise values, we found that surprising tones that were followed by erroneous probes were more surprising than those preceding correct WM, also with medium-to-large effect size (paired samples *t*-test, *N*=20, *t*(19)=2.14, *P*=0.045, *d*=0.77, Fig. 1c), which shows that WM failures in this task were directly related to the degree of surprise.

### Source-level EEG experiment

We then tested whether a neural suppressive process recruited by surprising events could explain the WM failures. In 20 new healthy volunteers, we recorded EEG for the WM task. We first replicated the behavioural pattern that WM accuracy was reduced followed surprising compared with standard tones (paired samples *t*-test, *N*=20, *t*(19)=2.3, *P*=0.033 , *d*=0.49). WM accuracy for the 2,200 interval was 80% for standard tones and 75.4% for novel tones; the significance test was based on that interval (WM accuracy for the three other intervals, which had standard tones only, was 80.6% for 1,700 ms, 81.3% for 2,700 ms and 81.7% for 3,200 ms). Again, surprising tones that were followed by WM failures were more surprising (paired samples *t*-test, *N*=20, *t*(19)=2.6, *P*=0.019, *d*=0.91). After the WM task, in the same session, we also recorded EEG during a version of the SST. In the SST (Fig. 1b), on each trial, participants initiated a response, which they had to try to rapidly stop if a visual stop signal occurred (this happened on 33% of trials). The stop-signal delay was adjusted online to ensure that stopping was sufficiently difficult^23^ and that successful and failed stop trials were of equal frequency. The behavioural pattern was typical for healthy young volunteers: The probability of successful stopping was 0.5, owing to the online adjustment of the stop-signal delay. Correct-go reaction time (RT) was 485 ms, and failed stop RT was significantly faster at 403 ms, as required by the race model of stopping^11^ (paired samples *t*-test, *N*=20, *t*(19)=12.81, *P*=8.5 × 10^−11^ , *d*=2; correct-go RT was greater than failed-stop RT in all participants). Stop-signal reaction time (SSRT), which indexes the speed of the stopping process, was 237 ms.

**Figure 2:**
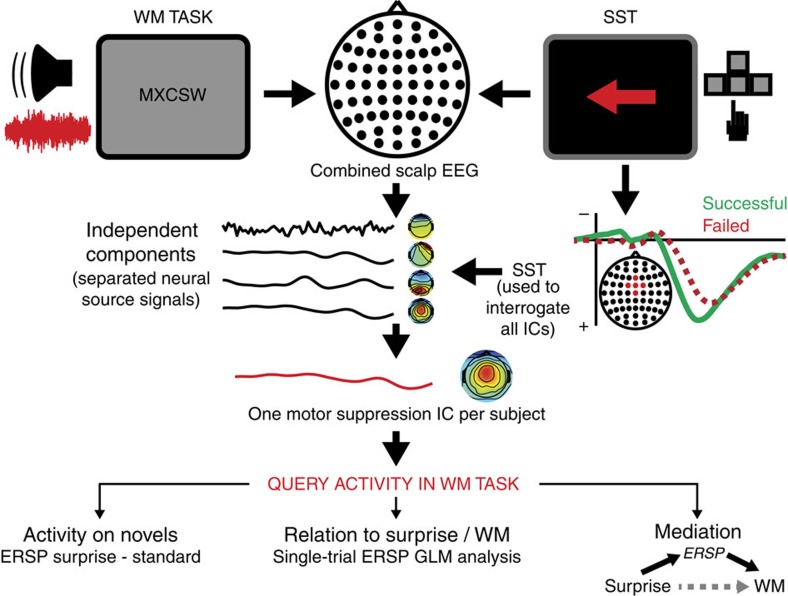
EEG analysis schematic. The combined EEG from the WM task and the SST were subjected to ICA. One IC that represented the process underlying successful stopping in the SST was selected per subject. The activity of that component in the WM task was then analysed in three separate ways: by showing that its activity was increased following surprising tones in the WM task, by showing that its activity was correlated with the trial-by-trial effects of surprise on WM accuracy, and by showing that its activity positively mediated between the surprise of the tone and accuracy on the WM probe.

In this experiment, we specifically used independent component analysis (ICA) to identify the brain source signal component that indexes successful motor stopping in the SST. For present purposes, we call this the ‘motor suppression-independent component' (MS-IC). We then aimed to investigate the activity of this MS-IC in the WM task. To achieve this, we computed an ICA on each participant's combined EEG from both tasks (Fig. 2). ICA decomposes the scalp-EEG mixture into its underlying independent source signal components (ICs), each of which represents an independent neural process that contributes to the scalp-EEG mixture^24^.

Crucially, the ICA logic allows us to test whether motor suppression in the SST and surprise-related activity in the WM task are independent processes or not: if motor suppression in the SST and surprise-related activity in the WM task are separable brain processes generated by independent sources, ICA on the combined data of both tasks will disentangle them into two separate ICs. In this scenario, any IC that is exclusively related to either motor suppression or surprise will show activity related to only one of the two events (i.e., successful stopping in the SST or surprising tones in the WM task). Conversely, any IC that is selected based on its activity related to one task event (e.g., successful stopping) but also shows significant activity following a different task event (e.g., surprising tones) must reflect activity of a common, non-separable brain mechanism represented by the same neural source (for other studies that use this inferential logic see refs 10, 25, 26, 27, 28, 29; for a review of the technique, see ref 29). It is important to note that this is not a null hypothesis test: this logic tests the alternative hypothesis that an independent component selected based on one type of experimental event also shows significantly increased activity following another, independent type of experimental event. Furthermore, it involves a purely data-driven procedure, in which an independent component is identified based on one part of the data (SST), and then investigated with respect to its activity on a separate part of the data that was not part of the selection process (WM task). Lastly, it allows us to test a specific hypothesis about the involvement of a clearly circumscribed neural process, in the absence of any variance related to processes that are unrelated to our *a priori* hypothesis.

**Figure 3:**
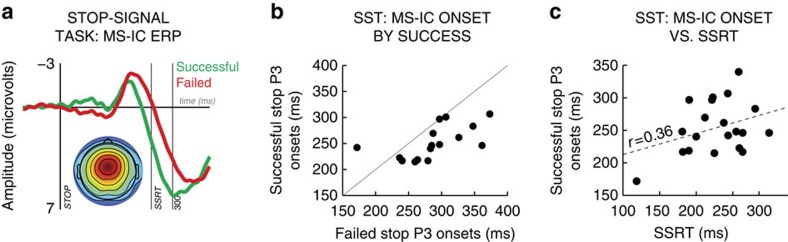
Source-level EEG results for the stop-signal task. (**a**) ERP back projection into channel space at component's centre of gravity. Inset: average topographical IC projection in channel space (rectified to show positive at FCz). (**b**) MS-IC ERP onset on successful and failed stop trials. Points represent participants. Four participants had no significant P3-ERP on failed stop trials (i.e., plot shows *N*=16). Points below the grey identity line demarcate participants whose MS-IC onset was earlier on successful compared to failed stop-signals (paired samples *t*-test, *N*=16, *t*(15)=3.62, *P*=0.003, *d*=0.77), which illustrates the relation of the MS-IC EEG activity to the speed of stopping. (**c**) Correlation between MC-IC ERP onset on successful stop trials and SSRT in *N*=20. Dashed line denotes least squares fit (*r*=0.36, *P*=0.06).

To apply this logic to our data set, we identified one source-level IC for each participant that represented the fronto-central P3-ERP (event-related potential) induced by the stop signal in the SST (see Supplementary Fig. 1 for complete ERP plots). Prior research has shown that this ERP specifically indexes the motor suppression process in the SST: its onset occurs in the precise time window when motor suppression is implemented^30^, correlates strongly with the speed of stopping (a large sample of *N*=62 revealed correlations between P3 onset and SSRT in excess of *r* >0.6^18^), and occurs earlier on trials on which stopping is successful^18^,^31^. Furthermore, the neural generators of this fronto-central component have been shown to be independent of other neural processes that are involved in the stop-signal task, such as those that reflect the attentional detection of the stop signal^19^. We selected one IC per subject that reflected this ERP (see Methods section), which we denote the MS-IC. To validate our MS-IC selection, we ensured that the onset of these selected source-level MS-ICs in our current data set showed the same tight relation to the success of stopping as prior studies (Fig. 3). Indeed, in line with prior studies^18^,^31^, the onset of the MS-IC P3-ERP occurred significantly earlier on successful stop trials compared with failed stop trials (paired samples *t*-test, *N*=16, *t*(15)=3.62, *P*=0.003, *d*=0.77, Fig. 3b), and was positively correlated with SSRT across participants (*r*=0.36, *P*=0.06, one-sided, Fig. 3c; outliers were diagnosed based on Cook's *d*>1, no outliers were present in the sample).

**Figure 4:**
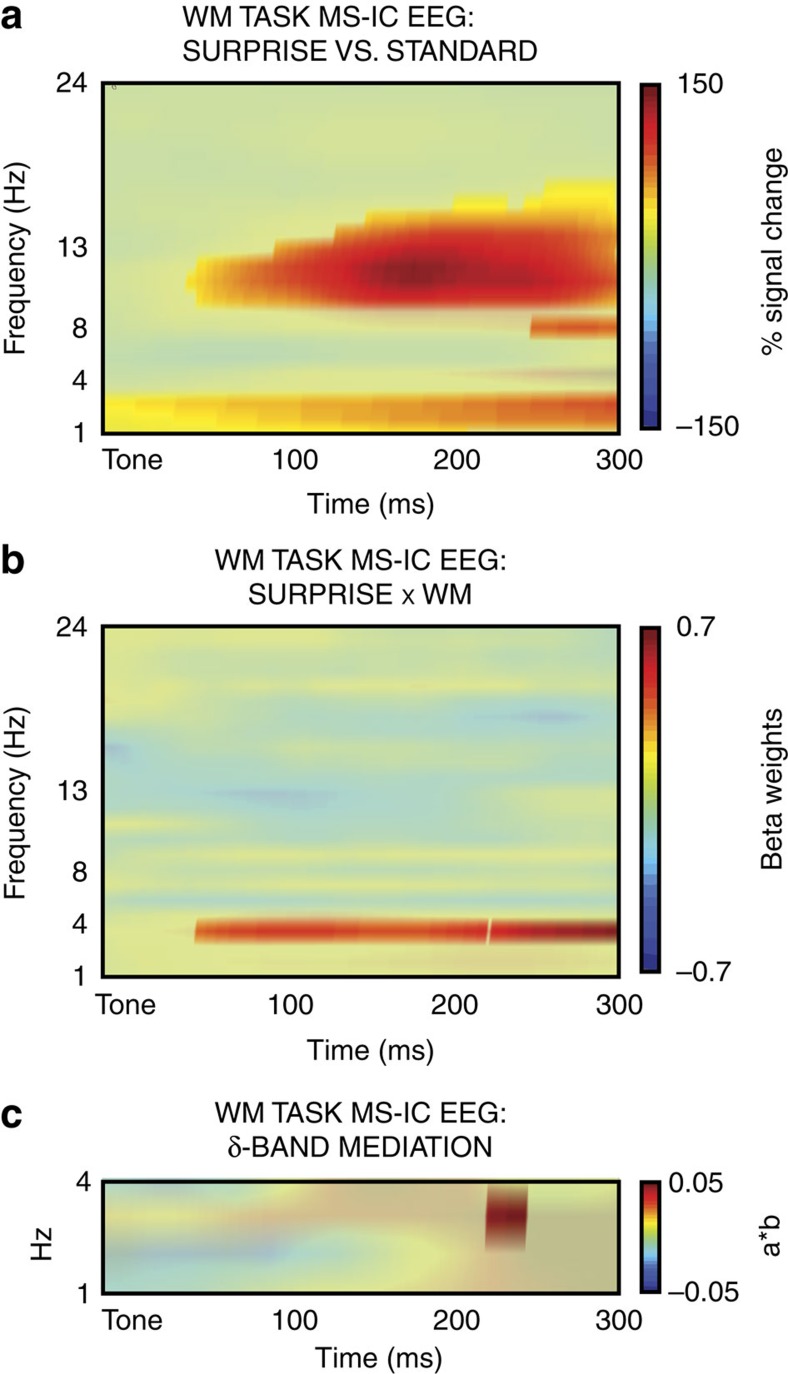
Source-level EEG results for the MS-IC WM task. (**a**) MS-IC ERSP following surprising versus standard tones in the WM task. Significant areas highlighted. The plot shows increased MS-IC activity for surprising tones in the WM task. (**b**) MS-IC single-trial GLM results from the WM task; group-average of the SURPRISE × WM interaction. Significant areas highlighted. (**c**) Mediation analysis. Areas in which ERSP significantly mediated surprise-related WM failures highlighted.

Following the MS-IC selection in each participant, we investigated its activity in the WM task. Hence, all the following analyses were performed on EEG source space, i.e., on independent component activity instead of scalp channel activity. We hypothesized, based on our prior study^10^ that the MS-ICs would be engaged following surprising events, indicated by increased 1–13 Hz activity within MS-ICs on surprising versus standard tones. This was indeed the case here (Fig. 4a). Hence, in line with our prior study^10^, we argue that the motor suppression process indexed by the MS-IC is active following surprising events (note that the MS-IC cannot reflect a process that is solely related to the surprise or infrequency of the tones, as it was identified based on its differential activity on successful versus failed stop trials in the SST. Stop-signals are not surprising, and successful and failed stop trials occur with equal frequency).

We then tested our main hypothesis of whether surprise-related MS-IC activity was related to WM failure using a single subject, single-trial general linear model (GLM). For each surprising tone, we quantified (a) the degree of SURPRISE of the tone derived from the Bayesian model (see Methods section), (b) the WM accuracy on the subsequent probe (1=failure, 0=no failure) and (c) the SURPRISE × WM interaction (this term has greater positive values if highly surprising tones are followed by WM failure). This GLM was applied to every single-trial sample point within the MS-ICs time–frequency source-level EEG response in the interval between surprising tones and the probe. This was done for all frequencies ranging from 1 to 24  Hz and all sample points from 1 to 300 ms following the tone individually. Standardized regression weights from these analyses were tested against zero on the group level to identify sections of the MS-ICs EEG that were significantly related to SURPRISE, WM or the SURPRISE × WM interaction. This analysis showed that delta-band (4 Hz) activity within the MS-ICs was positively related to the SURPRISE × WM interaction (Fig. 4b): on trials in which strong surprise was followed by subsequent WM failure, delta-band activity within MS-ICs was increased followed the surprising event. Supplementary Figure 2 shows that this correlation was largely driven by activity on incorrect novel trials, further supporting this association. No significant association with SURPRISE or WM was observed.

These results suggest a chain of events, in which surprise elicits delta-band activity in the MS-ICs, which in turn produces the WM failure. We tested this possible chain of events using a group-level mediation model (see Methods section; 6/20 participants did not have a sufficient number of trials for this analysis, leaving *N*=14). This analysis revealed that the influence of surprise on WM was indeed positively mediated by delta-band MS-IC activity (Fig. 4c). Put differently, the greater the amount of surprise on a trial, the greater was the WM disruption, which was mediated by EEG activity in the same brain system that is recruited to stop motor processes. These results confirm our earlier finding that a brain signature for outright motor stopping is also recruited by surprising events^10^, but strikingly, go much further by showing that the recruitment of this system leads to disruption of WM. We next focused on the STN of the basal ganglia.

### Intracranial STN-LFP experiment

Several studies have shown that outright motor stopping engages the STN^9^,^20^,^21^. As stated in the Introduction section, rapid motor stopping^12^,^13^,^14^,^15^, as well as surprising events^10^, have a broad suppressive effect on motor activity. We speculate that this broad suppressive effect could relate to the putative broad impact of the STN on the globus pallidus internus (GPi, also referred to as medial globus pallidus)^32^,^33^. We conjectured that since surprising tones engage the stopping system to interrupt WM (see above), surprise-related WM failure could relate to recruitment of the STN. In particular, we predicted that surprising tones evoke increased activity in STN, and that surprising tones that recruit the STN more heavily are more likely to be followed by WM failures, resulting in the same trial-to-trial SURPRISE × WM interaction we observed for the MS-IC in the scalp-EEG experiment.

To test this, we recorded LFPs from the STN during the WM task from seven Parkinson's disease patients who had depth electrodes implanted in the right STN. In each patient, we identified the contact that had the highest signal-to-noise ratio across all frequency bands (1–100 Hz) by comparing the analytic signal amplitude in the 300 ms following tone onset to a 100 ms pre-tone baseline, independent of trial type (i.e., for all trials, standards and surprising trials alike, see Methods section for more details).

**Figure 5:**
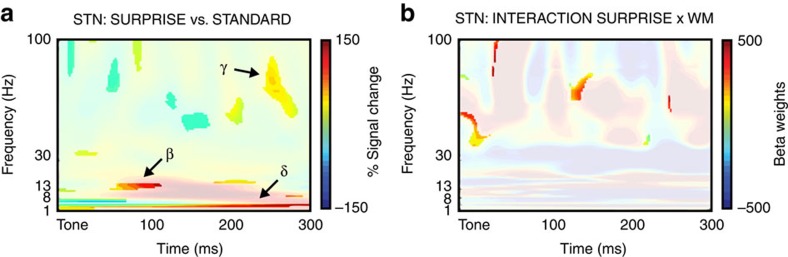
STN group results. (**a**) ERSP following surprising versus standard tones in the WM task. The plot shows increased MS-IC activity for surprising tones in the WM task. Significant areas highlighted. (**b**) Single-trial GLM results from the WM task; group-average of the SURPRISE × WM interaction. Significant areas highlighted.

We then analysed the LFP response in STN in the same way as the event-related spectral perturbation (ERSP) response of the MS-ICs in the scalp-EEG experiment, but with a broader frequency range (1–100 Hz), which included gamma activity. In line with our scalp-EEG results and our hypothesis, STN activity was increased following surprising events, notably in the delta (1–4 Hz), beta (13–30 Hz) and gamma (>30 Hz) frequency bands (Fig. 4a). The same single-subject GLM analysis as in the scalp-EEG experiment further showed that gamma-band activity was significantly related to the SURPRISE × WM interaction, just like the scalp MS-IC activity in the scalp-EEG experiment. Furthermore, single-subject plots (Fig. 5) show that each participant had significantly increased gamma-band activity in the STN following surprising tones (middle column of Fig. 5), and a significantly positive correlation with the SURPRISE × WM interaction (right column of Fig. 5). These results show that, just like the MS-IC scalp-EEG activity, LFP activity in the STN was significantly increased following surprising events. Furthermore and more importantly, just like the MS-IC scalp-EEG activity, LFP activity in the STN was significantly increased when stronger amounts of surprise lead to WM failure.

## Discussion

We tested whether a putative fronto-basal ganglia suppressive mechanism involved in motor stopping is recruited to interrupt cognitive activity (here, specifically, verbal WM) following surprising events. In a first experiment, we developed a behavioural paradigm in which surprising events led to WM failures. In a second experiment, we used source-level EEG to isolate a brain component that (we argue) reflects motor suppression in the SST. The results show that this same brain component was engaged when surprising events occurred during WM maintenance, and they furthermore suggest that its activity mediated the negative influence of surprise on WM. This was further corroborated by the third experiment, where we recorded LFPs through intracranial depth electrodes from the STN of the basal ganglia. We showed that activity in this key subcortical node of the motor suppression network was increased following surprising tones across several frequency bands, including the beta band, which has previously been proposed to be especially important for motor suppression^8^,^9^,^32^. Furthermore, STN gamma activity was increased when highly surprising tones were followed by WM failure. This modulatory gamma-band activity occurred within ∼150 ms following the tone, consistent with the timing of the broad corticomotor suppression that also occurs at 150 ms after surprising events^10^. We interpret these results as showing that a fronto-basal ganglia neural suppressive mechanism that broadly affects motor representations also affects cognition (here, verbal working memory). These findings widen the scope of a well-characterized neural system for motor suppression, provide new links between the literatures on response inhibition, working memory, surprise and attention, and also provide a mechanistic account of one type of distraction.

A key question is whether the ‘motor stopping' independent component (MS-IC) we identified in the SST truly reflects motor suppression, or rather low-level perceptual processing or attentional detection. While all of these three candidate processes are necessary for successful stopping in the stop-signal task, we argue that the MS-IC specifically reflects the suppressive process, i.e., the last process in this chain. This is based on the following reasoning. First, the neural mechanism reflected in the MS-IC cannot be low-level stimulus perception, since the MS-IC was active following both visual stop-signals and unexpected tones, which occurred in different sensory modalities. This leaves higher-level attentional detection and response suppression as candidate processes indexed by the MS-IC. The MS-IC was selected from our stop-signal data to reflect a fronto-central slow-wave (P3) potential, which has two key properties, as shown in previous studies and the current study (see refs 18, 31, and Fig. 3): its onset occurs earlier for successful compared with failed stop trials and it correlates with SSRT across subjects (SSRT marks the end of the stopping process). Previous studies have also shown that the onset of this fronto-central slow-wave component occurs in the time period immediately preceding SSRT (i.e., 10–20 ms before SSRT^18^). According to an influential model of the SST, this very late timing is a key characteristic of the motor suppression process^30^. Moreover, the late onset of this neural process in relation to SSRT makes it difficult to conceive that an additional process could realistically occur between this neural process and SSRT. Yet this would be necessary if the fronto-central slow-wave potential indexed attentional detection instead of motor suppression, since in that case, the actual motor suppression has yet to happen. Thus, these considerations strongly support our assertion that the MS-IC represents motor suppression. Further corroborating evidence comes from another study^19^, which showed that the fronto-central slow-wave potential explained gradual motor suppression in a complex visual stop-signal task, while attentional detection of a stop-signal, on the other hand, was indexed by a different independent signal component (a posterior component with a spectral peak in the alpha band). This shows that attentional detection maps onto an independent process that is markedly different from the fronto-central low-frequency potential we investigate in our current study, and is likely explained by a more posterior process with a different frequency characteristic (most likely in the alpha-band^33^).

The foregoing argues that the MS-IC component reflects motor suppression, and wider aspects of the data suggest the motor suppression process is responsible for the cognitive interruption observed here. Where does this leave stimulus-driven attention and attentional re-orienting, which also surely occur after unexpected tones in the WM task?^34^,^35^,^36^,^37^. To address this, we consider the relative timing of the individual processes. It has been shown that surprising tones can be perceptually detected in sensory areas as early as 50 ms after tone onset^38^. Studies with single-pulse transcranial magnetic stimulation (which has a time resolution at the millisecond level) have shown that the suppressive mechanism is active chronologically after this initial stage of low-level perceptual detection, namely, around 150 ms after the onset of the surprising tone^10^. Importantly, that study also showed the suppressive effect was transient, i.e., suppression was not observed at 175 or 200 ms post-tone. Therefore, the timing of the suppressive mechanism occurs well-before signatures of top-down attentional re-orienting, which usually emerge around 250–300 ms after a surprising event^39^,^40^,^41^. Hence, we propose the following model of how surprising events influence behaviour and cognition: After the initial perceptual detection of surprise (stimulus-driven attention, reflected in activity of early sensory areas), suppression is broadly exerted on both ongoing action and cognition (at least the verbal WM form of cognition studied here). This momentary suspension of ongoing cognitive and motor activity is then followed by attentional re-orienting. We suppose the suppressive mechanism has a useful function here, namely aiding the cognitive system to initiate an interrupt. This could enable a disengagement from ongoing motor and cognitive processes, in favour of enabling the rapid processing and evaluation of the surprising event (following an attentional shift). This model implies a quite different perspective from usual on attentional re-orienting. Here, re-orienting is only done *after* an interruption induced by a suppressive mechanism. This fits a recent proposal that attention is more of an effect than a cause^42^. More generally, this model also has implications for understanding disorders of distraction, such as attention-deficit hyperactivity disorder (in which perhaps the interruption is recruited too readily), and disorders of cognitive inflexibility, such as Parkinson's disease (in which perhaps the interruption does not occur readily enough).

By what neurobiological mechanism did the putative-STN-mediated response suppression system influence cognition in this study? Much research suggests that motor suppression is implemented via a hyperdirect pathway from frontal areas to the STN of the basal ganglia, which then activates GPi and suppresses thalamocortical drive^43^,^44^,^45^,^46^. The broad skeletomotor suppression that occurs for outright stopping^12^,^13^,^14^,^15^ could reflect the putative divergent innervation of the GPi by the STN, for which there is some evidence from neuronal tracing studies^47^,^48^,^49^. The engagement of this mechanism may also explain the broad skeletomotor suppression that occurs after surprising events^10^. Here, we show that surprising events also interrupt cognition via the same brain mechanism. One possibility is that the STN-mediated impact on GPi is so divergent as to also interrupt WM information that is putatively maintained in the ‘associative' cortico-basal ganglia loop^50^,^51^,^52^. We note however, that while this theory fits speculation that the STN has a massive impact, being at the ‘nexus of motor, limbic and associative processing'^53^,^54^,^55^, there is no direct evidence to our knowledge that one part of the STN can have such a divergent projection as to affect truly different sectors of the GPi (i.e., motor and associative). Instead, perhaps there is a divergent influence from STN to the motor GPi, enough to interrupt all motor-related processing, including, in this study, verbal WM, which is a form of cognition that is also motor based, being dependent on the language system^56^. Another possibility is that surprising events recruit several cortical areas that then project to different sectors of the STN (motor and associative), thus resulting in a broad suppressive effect (each one of the projections to the STN could then result in a relatively broad impact of that part of the STN on motor or associative sectors of GPi). Either way, our study provides evidence for a functional recruitment of the STN by surprising events, and it argues that this activity mediates the influence of surprise on cognition, although we warrant that exactly how this is done awaits further research. We also warrant that our hypothesis-driven ICA approach was non-exhaustive, i.e., it does not provide a complete picture of all commonalities and differences between surprise processing and action stopping. Instead, we focused from the outset on an IC that putatively reflects motor suppression, and tested whether its activity mediates the influence of surprise on WM. We acknowledge that there could be other surprise-related processes that we did not investigate here, which could further mediate or moderate this relationship. Still, our results suggest that the same suppressive mechanism recruited to stop action is recruited by surprise to disrupt WM. Future research is necessary to provide a more complete picture of the neural processes, and also to provide causal evidence that the putative-STN-mediated effect is responsible for the WM decrement.

In summary, surprising events manifest the same brain signature as outright stopping, and this mediates the effect of surprise on WM. This suppressive mechanism could interrupt thalamocortical processes in the same way it interrupts action, and allow the cognitive system to disengage from both behaviour and cognition. Such an interruption of ongoing cognition could facilitate (or even be necessary for) attentional re-orienting. This motivates a new neural systems theory of distraction, which is grounded in a cortico-basal ganglia network that underlies motor suppression, and can also affect cognition. These results broaden the scope of a well-characterized neural system for suppression, and they provide a critical link between response inhibition, working memory, surprise, and attention.

## Methods

### Participants

#### Behavioural experiment

Twenty-one people (mean age 20.5 years, s.e.m.: .37; two left handed; 14 female) participated in the behavioural experiment for course credit. Written informed consent was collected from all participants, and the study was approved by the UCSD Institutional Review Board.

#### Scalp-EEG experiment

Twenty-two people (mean age 23.2 years, s.e.m.: 1.73; one left handed; 12 female) participated in the EEG experiment for $15 per hour. Three participants (one behavioural, two EEG) were excluded because of sub-chance performance on the WM task.

#### Intracranial STN-LFP experiment

Seven people with Parkinson's disease (mean age 63.3 years, s.e.m.: 2.03; one left handed; four female) participated in the STN recording. It should be noted that participants were necessarily people with Parkinson's disease who had undergone neurosurgery. However, our aim was to record LFPs in as near as normal state as possible. To this end, patients were tested on their usual therapeutic medications (including dopamine replacement therapy) to best normalize their motor (and cognitive) capabilities.

### Working memory task

Each trial began with a string of consonants displayed in lowercase Times New Roman font (2,000 ms). Then, a fixation cross appeared for a maintenance interval (1,700, 2,200, 2,700 or 3,300 ms). Three-hundred millisecond before the end of the interval, a sound was played (200 ms). The sound was either a 600 Hz sine-wave tone (standard), or a unique birdsong segment; taken from ref. 10 (matched to the sine-wave in duration and volume). After the maintenance interval, a second string of letters (probe) was presented (uppercase Helvetica font). The probe was either the same as the first string (match), or one of the letters was exchanged for another consonant (no-match; probability: 0.5). The position of the exchanged letter was randomly drawn from a uniform distribution (however, the first letter was always the same as in the initial string). Participants had 2,000 ms to indicate whether the trial was a match or a no-match by pressing one of two keys. If no response was made, the trial was counted as a miss and a ‘Too slow!' message was displayed for 800 ms. Trials ended with an inter-trial interval (400 ms). Participants performed 180 trials in three blocks. Each block consisted of 12 standard trials (six match, six no-match) for each of the four delay interval durations, as well as 12 surprising trials (six match, six no-match; overall *p*(surprise)=0.2). Surprising trials always occurred after a 2,200 ms interval. Presenting all the surprising signals at the same time interval allowed us to employ a time-interval condition (2,200 ms) with an equal probability of standard versus novel trials, resulting in balanced trial numbers for all analyses presented in the manuscript, while still maintaining a low relative overall probability of surprising versus standard tones (0.2 versus 0.8), aided by the fact that participants could not anticipate the length of the maintenance interval on every trial. All analyses were conducted only on the surprising trials and the standard trials with the 2,200 ms delay period (i.e., 36 surprising and standard trials per participant), so as to not bias the behavioural analysis in case the task was is easier or harder at the 2,200 ms compared with the other time intervals. The tones were played at a normal conversational volume level, i.e., not loud enough to trigger a startle response. Trials were randomized in each block, except that the first three trials were always standard trials, and no two surprising trials could occur in immediate succession.

Before the experiment, the length of the string was determined in a training period (4 blocks of 12 standard trials in the behavioural experiment, 8 blocks in EEG); it was initially set to four (two for EEG). After each block of training, the length was reduced by one in case WM accuracy (proportion of hits on matches, proportion of correct rejections on no-matches) was below 60% for matches, no-matches or both. If accuracy exceeded 80% for both matches and no-matches, the length was increased. This was done in order to make the task sufficiently difficult, but to avoid sub-chance performance.

### Behavioural analysis

Paired samples *t*-tests were used to assess the influence of trial type on WM accuracy. Surprise was quantified as follows:


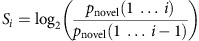


This equation uses the Kullback–Leibler divergence between the posterior probability of a surprising tone (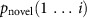, i.e., the probability of a surprising tone on all trials up onto and including the current trial) and the prior expectation of a surprising tone (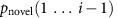, i.e., the probability of a surprising tone on all trials up onto - *but excluding* - the current trial). This results in values between 0 and 1, except for the first surprising trials (value not defined), for which we set the value to 1. (We assume here that individual birdsong segments were equally surprising. This is certainly only approximately correct. What is important is that the birdsong segments were of equal volume envelope and duration, and their order was randomized across subjects.) We then compared these values for correct and incorrect surprising trials using paired samples *t*-tests. Note that this variable is calculated truly independently of behaviour and brain activity. Effect sizes were expressed in units of Cohen's d.

### Stop-signal task

Trials began with a fixation cross (500 ms), followed by a left- or right-ward arrow (go-stimulus). Participants had to respond as fast and accurately as possible to the arrow using their right index or middle finger. On 33% of trials, a stop signal occurred (the arrow turned from white to red) at a delay after the go-stimulus (stop-signal delay, SSD). The SSD (initially 200 ms) was dynamically adjusted (in 50 ms increments) to achieve a *p*(stop) of 0.5: after successful stops, SSD was prolonged; after failed stops, it was shortened (independently for left- and rightward go-stimuli). Trial duration was fixed (3,000 ms). Six blocks of 50 trials were performed.

### Scalp-EEG recording

EEG was recorded using a 64-channel system (BioSemi Instrumentation, 512 Hz sampling rate), plus eight electrodes placed on the bilateral mastoids and around the eyes, in a copper-shielded, sound attenuated chamber. Stimuli were presented on a CRT-monitor (NEC FB2141SB). The data were on-line referenced to the CMS-DRL reference, all reference-offsets were kept below 25 μV. Participants performed the WM task first and the SST second (to not bias them towards using the stopping-network in the WM task).

### EEG preprocessing

Data were preprocessed using custom routines in MATLAB 2012a (MathWorks). ICA was performed using functions from the EEGLAB toolbox^57^. Preprocessing was identical to our previous studies, see ref. 10 for details.

### Selection of motor suppression-independent component

We recently showed that the fronto-central source-level P3-ERP component time locked to the stop signal is an index of the motor suppression process in the SST^18^. Hence, we selected one component per participant that represented the neural source process underlying this ERP. To do so, we first selected each component whose weight matrix had its maximal rectified weight at one of the fronto-central electrodes (Fz, FCz, Cz, FC1 and FC2). We then averaged those components' back-projected channel-space activity at these fronto-central electrodes within the 500 ms time period following the stop signal, and correlated this event-related average activity to the event-related average activity of the overall EEG data (i.e., the EEG data based on the back projection of all non-artifact ICs for that participant) in that time range. The component that showed the highest correlation with the overall ERP was selected as the MS-IC.

### Event-related spectral perturbation

We calculated the ERSP (i.e., the time–frequency response) of the MS-IC following both surprising and standard trials (1 to 50±0.5 Hz, linearly spaced) using the absolute of the Hilbert transform as an analytic signal for each frequency (converted into %-change compared with a 500 ms pre-tone baseline). We tested this ERSP for significance on the group-level using individual *t*-tests of surprising versus standard trial activity at each time × frequency sample point, resulting in 153 × 50=7650 individual tests. The significance level of *P*<0.05, was corrected using the FDR method^58^ to a critical *P* value of 0.0159.

### EEG single-trial GLM analysis

To investigate the relation between the MS-IC ERSP and SURPRISE, WM accuracy (and the SURPRISE × WM interaction) after surprising tones, we constructed individual GLMs for each participant. WM accuracy on each surprising trial was coded with 1 for misses or false alarms, and 0 for hits or correct rejections. Surprise was computed as above.

Both variables were de-meaned, and then their interaction was calculated by multiplication. De-meaning controls for the fact that surprise and WM failure are correlated, and results in a valid regression model (the variance inflation factor, VIF, for each condition was below 10 in all participants except 3 [mean VIF=1.76]; excluding these three participants did not significantly alter the results).

These three variables were used as regressors to model each individual time × frequency sample point of the tone-related MS-IC ERSP on each surprising trial. The standardized beta weights of these regression models were then tested for significance on the group-level using individual Wilcoxon signed-rank tests from 0 for each time × frequency sample point. Again, this resulted in 7,650 tests; the significance level of *P*<0.05 was FDR-adjusted to 0.0004.

### EEG single-trial mediation analysis

To test whether the tone-related delta-band MS-IC ERSP mediated between SURPRISE and WM, we performed a mediation analysis for dichotomous outcome variables^59^,^60^. For this, we constructed two logistic regression models for each participant. In one model, SURPRISE was modelled onto each individual sample point of the tone-related MS-IC ERSP at each delta-band (1–4 Hz) frequency (value ‘*a*' in Baron & Kenny's model). In a second model, the single-trial MS-IC ERSP was modelled onto WM failure (outcome variable) using a logistic regression. Crucially, this was done while controlling for the effect of surprise on WM failure by including both the MS-IC ERSP, as well as the surprise regressor into this model. The resulting regression weights for the ERSP regressor denote value ‘*b*' in Baron & Kenny's model (all parametric terms that went into these analyses were standardized to *z*-scores).

The mediating influence of the ERSP response of the MS-IC at each individual time–frequency point is then expressed as the product of *a* × *b* (i.e., the mediation effect or ‘indirect path' in Baron & Kenny's model). This product was generated for each individual time–frequency point within 300 ms of the onset of the surprising tone for all delta-frequencies in each individual participant. The resultant values for the product of *a* × *b* were then tested against zero on the group-level using Wilcoxon signed-rank tests (*P*=0.05, one-sided to test for significant positive mediation). This analysis was restricted to the previous frequency range of interest (delta-band), on account of our very strong a priori hypothesis based on the GLM and ERSP analyses. With regards to the time range, we show the entire 300 ms post-tone time range in our plot (Fig. 4c) to avoid complicating the display.

Six out of the 20 participants did not have a sufficient number of trials for a valid logistic regression analysis (inclusion: minimum of five failed WM trials), leaving a remaining sample of *N*=14 for this analysis.

### Intracranial data preprocessing

Individual electrode contacts were re-referenced according to a dipolar nearest neighbour reference. Data were downsampled to 500 Hz, filtered between 0.5 and 100 Hz using zero-phase finite impulse response filters, and visually checked for stretches of data with artifact contamination, which were removed from further analysis. Based on visual inspection, we removed between 22 and 53 s of data (out of ∼20 min) for each participant (mean: 35.4 s).

### STN electrode contact selection

Six patients had four contacts in right STN, while one had eight. For each participant, we selected the bipolar contact with the highest signal-to-noise ratio across the entire ERSP frequency spectrum in the first 300 ms following the tone, regardless of trial type. To this end, the root mean square of the absolute of the Hilbert transformed signal was quantified in the first 300 ms following tone onset, and was divided by the root mean square of the absolute of the Hilbert transformed signal in the 100 ms before tone onset. The contact with the highest ratio across all trials was selected for further analysis.

### LFP data analysis

**Figure 6:**
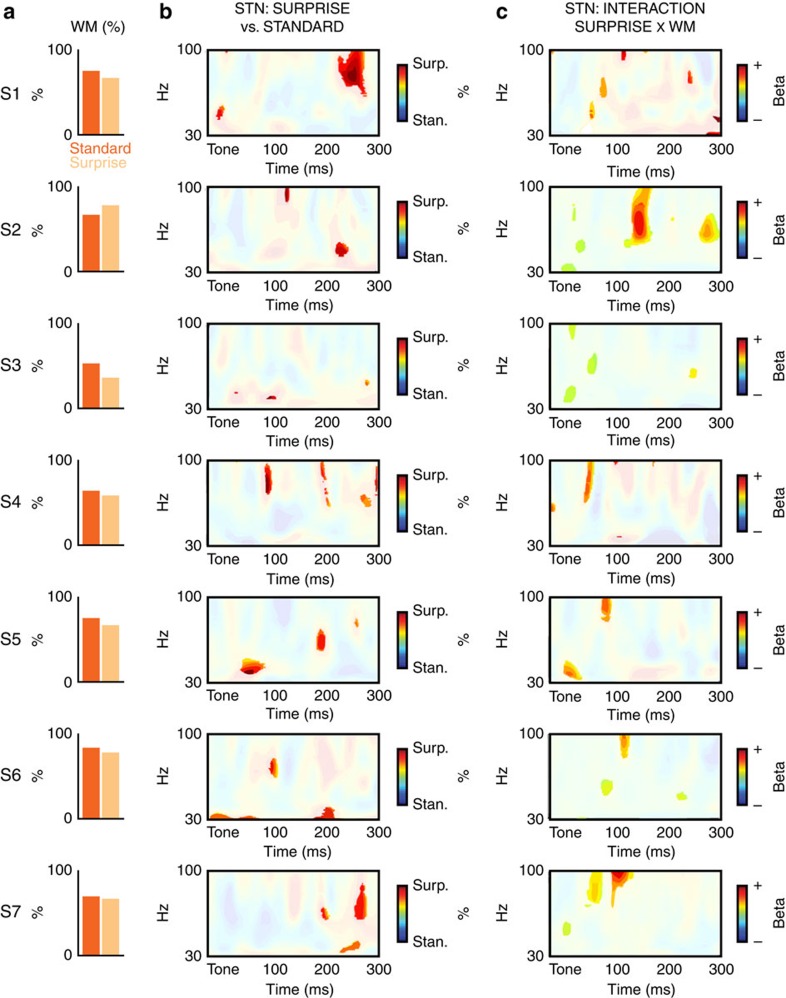
STN individual subjects results. (**a**) Behavioural results (accuracy on the WM probe split by trial type). (**b**) Individual contrasts of surprising versus standard events, specifically in the gamma band (significant areas at *P*<0.01 highlighted). (**c**) Individual single-trial GLM results, specifically in the gamma band (significant areas at *P*<0.05 highlighted).

ERSPs were computed as for scalp-EEG above (with a broader frequency spectrum of 1–100 Hz), thresholded for significance at *P*<0.05 on the group level. For the individual subjects data figure (Fig. 6), significance at *P*<0.01 is displayed. The GLM analysis was also performed as it was for the scalp-EEG (with a broader frequency spectrum of 1–100 Hz), thresholded for significance at *P*<0.05.

## Additional information

**How to cite this article:** Wessel, J. R. *et al.* Surprise disrupts cognition via a fronto-basal ganglia suppressive mechanism. *Nat. Commun.* 7:11195 doi: 10.1038/ncomms11195 (2016).

## Supplementary Material

Supplementary Information Supplementary Figures 1-2

## References

[b1] Nachev P., Kennard C. & Husain M. Functional role of the supplementary and pre-supplementary motor areas. Nat. Rev. Neurosci. 9, 856–869 (2008). 18843271 10.1038/nrn2478

[b2] Duann J. R., Ide J. S., Luo X. & Li C. S. Functional connectivity delineates distinct roles of the inferior frontal cortex and presupplementary motor area in stop signal inhibition. J. Neurosci. 29, 10171–10179 (2009). 19675251 10.1523/JNEUROSCI.1300-09.2009 PMC2769086

[b3] Wessel J. R., Conner C. R., Aron A. R. & Tandon N. Chronometric electrical stimulation of right inferior frontal cortex increases motor braking. J. Neurosci. 33, 19611–19619 (2013). 24336725 10.1523/JNEUROSCI.3468-13.2013 PMC3858630

[b4] Aron A. R., Fletcher P. C., Bullmore E. T., Sahakian B. J. & Robbins T. W. Stop-signal inhibition disrupted by damage to right inferior frontal gyrus in humans. Nat. Neurosci. 6, 115–116 (2003). 12536210 10.1038/nn1003

[b5] Whelan R. *et al.* Adolescent impulsivity phenotypes characterized by distinct brain networks. Nat. Neurosci. 15, 920–925 (2012). 22544311 10.1038/nn.3092

[b6] Swann N. *et al.* Intracranial EEG reveals a time- and frequency-specific role for the right inferior frontal gyrus and primary motor cortex in stopping initiated responses. J. Neurosci. 29, 12675–12685 (2009). 19812342 10.1523/JNEUROSCI.3359-09.2009 PMC2801605

[b7] Aron A. R. & Poldrack R. A. Cortical and subcortical contributions to Stop signal response inhibition: role of the subthalamic nucleus. J. Neurosci. 26, 2424–2433 (2006). 16510720 10.1523/JNEUROSCI.4682-05.2006 PMC6793670

[b8] Kuhn A. A. *et al.* Event-related beta desynchronization in human subthalamic nucleus correlates with motor performance. Brain 127, 735–746 (2004). 14960502 10.1093/brain/awh106

[b9] Ray N. J. *et al.* The role of the subthalamic nucleus in response inhibition: evidence from local field potential recordings in the human subthalamic nucleus. Neuroimage 60, 271–278 (2012). 22209815 10.1016/j.neuroimage.2011.12.035

[b10] Wessel J. R. & Aron A. R. Unexpected events induce motor slowing via a brain mechanism for action-stopping with global suppressive effects. J. Neurosci. 33, 18481–18491 (2013). 24259571 10.1523/JNEUROSCI.3456-13.2013 PMC3834054

[b11] Logan G. D., Cowan W. B. & Davis K. A. On the ability to inhibit simple and choice reaction-time responses - a model and a method. J. Exp. Psychol. Hum. Percept. Perform. 10, 276–291 (1984). 6232345 10.1037//0096-1523.10.2.276

[b12] Badry R. *et al.* Suppression of human cortico-motoneuronal excitability during the Stop-signal task. Clin. Neurophysiol. 120, 1717–1723 (2009). 19683959 10.1016/j.clinph.2009.06.027

[b13] Majid D. S., Cai W., George J. S., Verbruggen F. & Aron A. R. Transcranial magnetic stimulation reveals dissociable mechanisms for global versus selective corticomotor suppression underlying the stopping of action. Cereb. Cortex 22, 363–371 (2012). 21666129 10.1093/cercor/bhr112 PMC3256406

[b14] Wessel J. R., Reynoso H. S. & Aron A. R. Saccade suppression exerts global effects on the motor system. J. Neurophysiol. 110, 883–890 (2013). 23699058 10.1152/jn.00229.2013 PMC3742979

[b15] Cai W., Oldenkamp C. L. & Aron A. R. Stopping speech suppresses the task-irrelevant hand. Brain Lang. 120, 412–415 (2012). 22206872 10.1016/j.bandl.2011.11.006 PMC3533487

[b16] Wessel J. R., O'Doherty J. P., Berkebile M. M., Lindermann D. & Aron A. R. Stimulus devaluation induced by stopping action. J. Exp. Psychol. Gen. 143, 2316–2329 (2014). 25313953 10.1037/xge0000022 PMC4244281

[b17] Chiu Y. C. & Egner T. Inhibition-induced forgetting: when more control leads to less memory. Psychol. Sci. 26, 27–38 (2015). 25398560 10.1177/0956797614553945 PMC4353579

[b18] Wessel J. R. & Aron A. R. It's not too late: the onset of the frontocentral P3 indexes successful response inhibition in the stop-signal paradigm. Psychophysiology 52, 472–480 (2014). 25348645 10.1111/psyp.12374 PMC4830357

[b19] Wessel J. R. & Aron A. R. Inhibitory motor control based on complex stopping goals relies on the same brain network as simple stopping. Neuroimage 103C, 225–234 (2014). 25270603 10.1016/j.neuroimage.2014.09.048 PMC4396635

[b20] Bastin J. *et al.* Inhibitory control and error monitoring by human subthalamic neurons. Transl. Psychiatry 4, e439 (2014). 25203170 10.1038/tp.2014.73 PMC4203004

[b21] Alegre M. *et al.* The subthalamic nucleus is involved in successful inhibition in the stop-signal task: a local field potential study in Parkinson's disease. Exp. Neurol. 239, 1–12 (2013). 22975442 10.1016/j.expneurol.2012.08.027

[b22] Aron A. R., Behrens T. E., Smith S., Frank M. J. & Poldrack R. A. Triangulating a cognitive control network using diffusion-weighted magnetic resonance imaging (MRI) and functional MRI. J. Neurosci. 27, 3743–3752 (2007). 17409238 10.1523/JNEUROSCI.0519-07.2007 PMC6672420

[b23] Verbruggen F. & Logan G. D. Models of response inhibition in the stop-signal and stop-change paradigms. Neurosci. Biobehav. Rev. 33, 647–661 (2009). 18822313 10.1016/j.neubiorev.2008.08.014 PMC2696813

[b24] Onton J., Westerfield M., Townsend J. & Makeig S. Imaging human EEG dynamics using independent component analysis. Neurosci. Biobehav. Rev. 30, 808–822 (2006). 16904745 10.1016/j.neubiorev.2006.06.007

[b25] Wessel J. R., Danielmeier C., Morton J. B. & Ullsperger M. Surprise and error: common neuronal architecture for the processing of errors and novelty. J. Neurosci. 32, 7528–7537 (2012). 22649231 10.1523/JNEUROSCI.6352-11.2012 PMC6703591

[b26] Torrecillos F., Albouy P., Brochier T. & Malfait N. Does the processing of sensory and reward-prediction errors involve common neural resources? Evidence from a frontocentral negative potential modulated by movement execution errors. J. Neurosci. 34, 4845–4856 (2014). 24695704 10.1523/JNEUROSCI.4390-13.2014 PMC6802716

[b27] van Noordt S. J., Desjardins J. A. & Segalowitz S. J. Watch out! Medial frontal cortex is activated by cues signaling potential changes in response demands. Neuroimage 114, 356–370 (2015). 25887260 10.1016/j.neuroimage.2015.04.021

[b28] Gentsch A., Ullsperger P. & Ullsperger M. Dissociable medial frontal negativities from a common monitoring system for self- and externally caused failure of goal achievement. Neuroimage 47, 2023–2030 (2009). 19486945 10.1016/j.neuroimage.2009.05.064

[b29] Wessel J. R. Testing Multiple Psychological Processes for Common Neural Mechanisms Using EEG and Independent Component Analysis. Brain Topography (in press). DOI: 10.1007/s10548-016-0483-5 . 10.1007/s10548-016-0483-5 26956562

[b30] Boucher L., Palmeri T. J., Logan G. D. & Schall J. D. Inhibitory control in mind and brain: an interactive race model of countermanding saccades. Psychol. Rev. 114, 376 (2007). 17500631 10.1037/0033-295X.114.2.376

[b31] Kok A., Ramautar J. R., De Ruiter M. B., Band G. P. & Ridderinkhof K. R. ERP components associated with successful and unsuccessful stopping in a stop-signal task. Psychophysiology 41, 9–20 (2004). 14692996 10.1046/j.1469-8986.2003.00127.x

[b32] Swann N. *et al.* Deep brain stimulation of the subthalamic nucleus alters the cortical profile of response inhibition in the beta frequency band: a scalp EEG study in Parkinson's disease. J. Neurosci. 31, 5721–5729 (2011). 21490213 10.1523/JNEUROSCI.6135-10.2011 PMC3086079

[b33] Yamagishi N. *et al.* Attentional modulation of oscillatory activity in human visual cortex. Neuroimage 20, 98–113 (2003). 14527573 10.1016/s1053-8119(03)00341-0

[b34] Corbetta M., Patel G. & Shulman G. L. The reorienting system of the human brain: from environment to theory of mind. Neuron 58, 306–324 (2008). 18466742 10.1016/j.neuron.2008.04.017 PMC2441869

[b35] Corbetta M. & Shulman G. L. Control of goal-directed and stimulus-driven attention in the brain. Nat. Rev. Neurosci. 3, 201–215 (2002). 11994752 10.1038/nrn755

[b36] Menon V. & Uddin L. Q. Saliency, switching, attention and control: a network model of insula function. Brain Struct. Funct. 214, 655–667 (2010). 20512370 10.1007/s00429-010-0262-0 PMC2899886

[b37] Levy B. J. & Wagner A. D. Cognitive control and right ventrolateral prefrontal cortex: reflexive reorienting, motor inhibition, and action updating. Ann. N. Y. Acad. Sci. 1224, 40–62 (2011). 21486295 10.1111/j.1749-6632.2011.05958.x PMC3079823

[b38] Gamble M. L. & Woldorff M. G. The temporal cascade of neural processes underlying target detection and attentional processing during auditory search. Cereb. Cortex 25, 2456–2465 (2014). 24711486 10.1093/cercor/bhu047 PMC4537419

[b39] Barcelo F., Perianez J. A. & Knight R. T. Think differently: a brain orienting response to task novelty. Neuroreport 13, 1887–1892 (2002). 12395085 10.1097/00001756-200210280-00011

[b40] Donchin E. Presidential address, 1980. Surprise!...Surprise? Psychophysiology 18, 493–513 (1981). 7280146 10.1111/j.1469-8986.1981.tb01815.x

[b41] SanMiguel I., Morgan H. M., Klein C., Linden D. & Escera C. On the functional significance of Novelty-P3: facilitation by unexpected novel sounds. Biol. Psychol. 83, 143–152 (2010). 19963034 10.1016/j.biopsycho.2009.11.012

[b42] Krauzlis R. J., Bollimunta A., Arcizet F. & Wang L. Attention as an effect not a cause. Trends Cogn. Sci. 18, 457–464 (2014). 24953964 10.1016/j.tics.2014.05.008 PMC4186707

[b43] Aron A. R. From reactive to proactive and selective control: developing a richer model for stopping inappropriate responses. Biol. Psychiatry 69, e55–e68 (2011). 20932513 10.1016/j.biopsych.2010.07.024 PMC3039712

[b44] Bari A. & Robbins T. W. Inhibition and impulsivity: behavioral and neural basis of response control. Prog. Neurobiol. 108, 44–79 (2013). 23856628 10.1016/j.pneurobio.2013.06.005

[b45] Schmidt R., Leventhal D. K., Mallet N., Chen F. & Berke J. D. Canceling actions involves a race between basal ganglia pathways. Nat. Neurosci. 16, 1118–1124 (2013). 23852117 10.1038/nn.3456 PMC3733500

[b46] Isoda M. & Hikosaka O. Role for subthalamic nucleus neurons in switching from automatic to controlled eye movement. J. Neurosci. 28, 7209–7218 (2008). 18614691 10.1523/JNEUROSCI.0487-08.2008 PMC2667154

[b47] Nauta H. J. & Cole M. Efferent projections of the subthalamic nucleus. Trans. Am. Neurol. Assoc. 99, 170–173 (1974). 4142821

[b48] Smith Y., Hazrati L. N. & Parent A. Efferent projections of the subthalamic nucleus in the squirrel monkey as studied by the PHA-L anterograde tracing method. J. Comp. Neurol. 294, 306–323 (1990). 2332533 10.1002/cne.902940213

[b49] Nauta H. J. & Cole M. Efferent projections of the subthalamic nucleus: an autoradiographic study in monkey and cat. J. Comp. Neurol. 180, 1–16 (1978). 418083 10.1002/cne.901800102

[b50] Hazy T. E., Frank M. J. & O'Reilly R. C. Towards an executive without a homunculus: computational models of the prefrontal cortex/basal ganglia system. Philos. Trans. R. Soc. Lond. B Biol. Sci. 362, 1601–1613 (2007). 17428778 10.1098/rstb.2007.2055 PMC2440774

[b51] Chatham C. H., Frank M. J. & Badre D. Corticostriatal output gating during selection from working memory. Neuron 81, 930–942 (2014). 24559680 10.1016/j.neuron.2014.01.002 PMC3955887

[b52] McNab F. & Klingberg T. Prefrontal cortex and basal ganglia control access to working memory. Nat. Neurosci. 11, 103–107 (2008). 18066057 10.1038/nn2024

[b53] Baunez C., Yelnik J. & Mallet L. Six questions on the subthalamic nucleus: lessons from animal models and from stimulated patients. Neuroscience 198, 193–204 (2011). 22001680 10.1016/j.neuroscience.2011.09.059

[b54] Gillies A. J. & Willshaw D. J. A massively connected subthalamic nucleus leads to the generation of widespread pulses. Proc. Biol. Sci. 265, 2101–2109 (1998). 9842737 10.1098/rspb.1998.0546 PMC1689499

[b55] Rektor I., Bockova M., Chrastina J., Rektorova I. & Balaz M. The modulatory role of subthalamic nucleus in cognitive functions - a viewpoint. Clin. Neurophysiol. 126, 653–658 (2015). 25487910 10.1016/j.clinph.2014.10.156

[b56] Morra S. How do subvocal rehearsal and general attentional resources contribute to verbal short-term memory span? Front. Psychol. 6, 145 (2015). 25798114 10.3389/fpsyg.2015.00145 PMC4351569

[b57] Delorme A. & Makeig S. EEGLAB: an open source toolbox for analysis of single-trial EEG dynamics including independent component analysis. J. Neurosci. Methods 134, 9–21 (2004). 15102499 10.1016/j.jneumeth.2003.10.009

[b58] Benjamini Y. & Hochberg Y. Controlling the false discovery rate - a practical and powerful approach to multiple testing. J. R. Statist. Soc. B 57, 289–300 (1995).

[b59] Baron R. M. & Kenny D. A. The moderator mediator variable distinction in social psychological-research - conceptual, strategic, and statistical considerations. J. Pers. Soc. Psychol. 51, 1173–1182 (1986). 3806354 10.1037//0022-3514.51.6.1173

[b60] Mackinnon D. P. & Dwyer J. H. Estimating mediated effects in prevention studies. Eval. Rev. 17, 144–158 (1993).

