# Supplementary material for: Surprise disrupts cognition via a fronto-basal ganglia suppressive mechanism
Source: Nat Commun. 2016 Apr 18;7:11195. doi: 10.1038/ncomms11195 (PMC4837448; doi:10.1038/ncomms11195)
Supplement: Supplementary Information — Supplementary Figures 1-2 [file ncomms11195-s1.pdf]

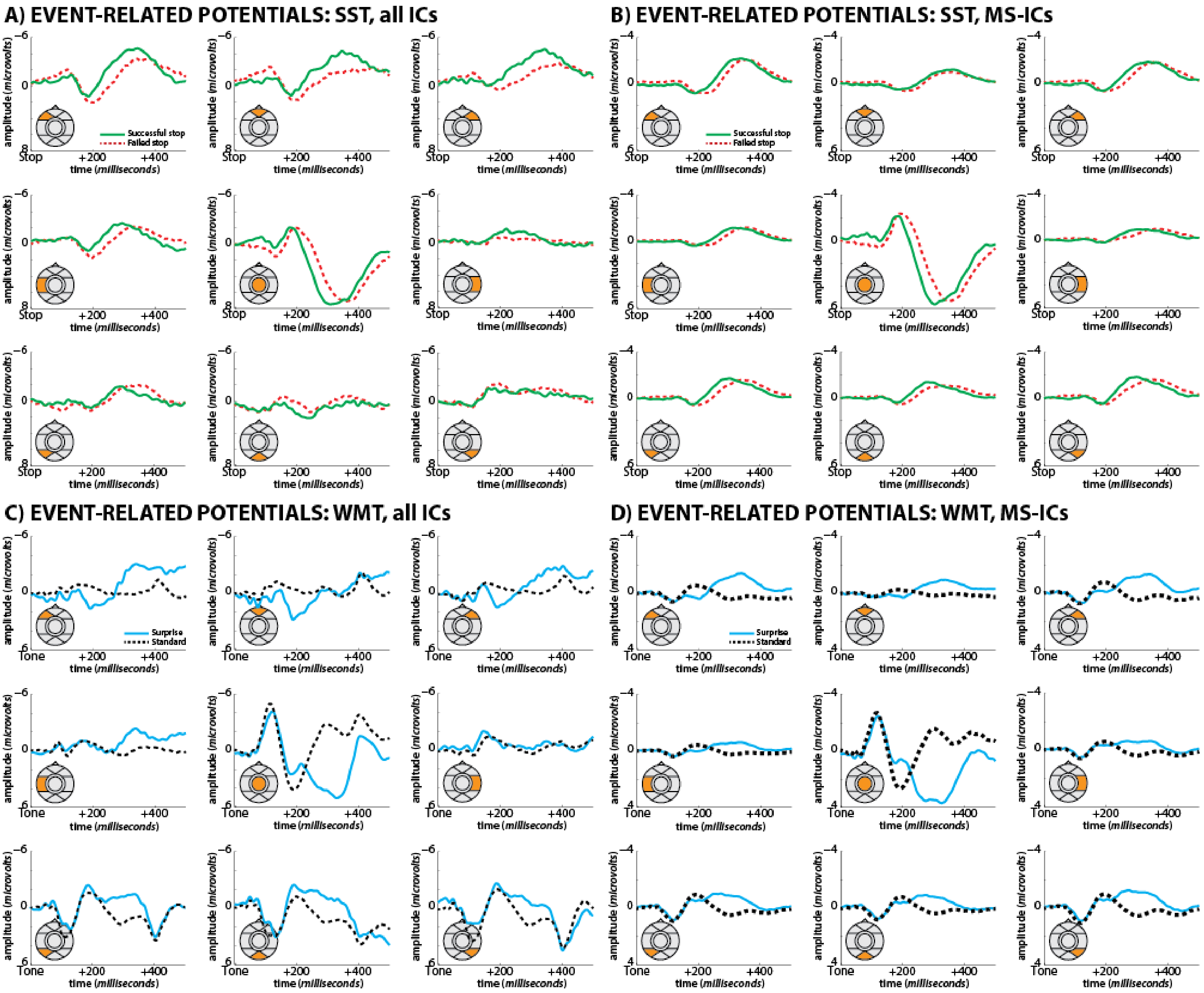

Supplementary Figure 1. Event-related potential time courses from Experiment 2. A) Stop-signal locked ERPs from the stop-signal task, based on all independent components (excluding artifacts, i.e., eye-blinks etc.). B) Stop-signal locked ERPs from the stop-signal task, based on the MS-ICs alone. C) Tone-locked ERPs from the working memory task, based on all independent components (excluding artifacts, i.e., eye-blinks etc.). D) Tone-locked ERPs from the working memory task, based on the MS-ICs alone.

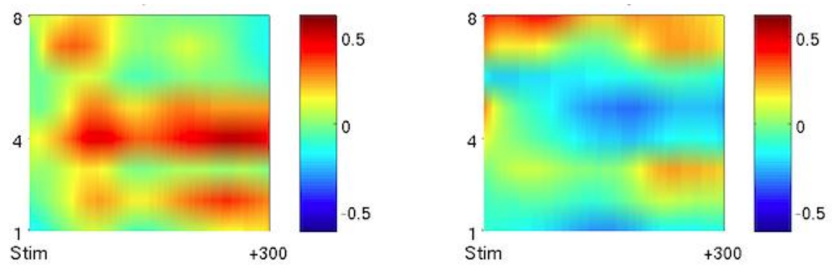

Supplementary Figure 2. These panels show the correlation between tone-related ERSP activity in the WM task and the SURPRISE regressor, separately for incorrect WM trials (left panel) and correct WM trials (right panel). As can be seen, the positive correlation between the SURPRISE * WM interaction of the overall GLM and the tone-related ERSP activity (~4Hz, see Figure 4B), is largely explained by the incorrect WM trials.
